# High expression of RelA/p65 is associated with activation of nuclear factor-*κ*B-dependent signaling in pancreatic cancer and marks a patient population with poor prognosis

**DOI:** 10.1038/sj.bjc.6603878

**Published:** 2007-07-10

**Authors:** W Weichert, M Boehm, V Gekeler, M Bahra, J Langrehr, P Neuhaus, C Denkert, G Imre, C Weller, H-P Hofmann, S Niesporek, J Jacob, M Dietel, C Scheidereit, G Kristiansen

**Affiliations:** 1 Institute of Pathology, Charité University Hospital, Berlin, Germany; 2 Department Pharmacology-Oncology, ALTANA Pharma AG, Konstanz, Germany; 3 Department of General, Visceral, and Transplantation Surgery, Charité University Hospital, Berlin, Germany; 4 Max-Delbrück-Center for Molecular Medicine, Berlin, Germany

**Keywords:** NF-*κ*B, pancreatic carcinoma, RelA, prognosis, survival, immunohistochemistry

## Abstract

Activation of nuclear factor-*κ*B (NF-*κ*B) signaling was observed in pancreatic adenocarcinoma cell lines and tumours. However, information on the expression of RelA/p65, the major transcription activating NF-*κ*B subunit, in these carcinomas and possible correlations thereof with NF-*κ*B activation and patient survival is not available. To provide this missing translational link, we analysed expression of RelA/p65 in 82 pancreatic adenocarcinomas by immunohistochemistry. Moreover, we measured activation of the NF-*κ*B pathway in 11 tumours by quantitative PCR for NF-*κ*B target genes. We observed strong cytoplasmic or nuclear expression of RelA/p65 in 42 and 37 carcinomas, respectively. High cytoplasmic and nuclear expression of RelA/p65 had negative prognostic impact with 2-year survival rates for patients without cytoplasmic or nuclear RelA/p65 positivity of 41 and 40% and rates for patients with strong cytoplasmic or nuclear RelA/p65 expression of 22 and 20%, respectively. High RelA/p65 expression was correlated to increased expression of NF-*κ*B target genes. The observation that high expression of RelA/p65 is correlated to an activation of the NF-*κ*B pathway and indicates poor patient survival identifies a patient subgroup that might particularly benefit from NF-*κ*B-inhibiting agents in the treatment of pancreatic cancer. Based on our findings, this subgroup could be identified by applying simple immunohistochemical techniques.

Pancreatic carcinoma is one of the most aggressive of all cancer types, in which the mortality rate almost equals the rate of incidence (Jemal *et al*, 2006). The clinically silent onset and the lack of an effective therapy for advanced stage tumours contribute substantially to the deleterious 5-year survival rate of less than 5% (Mimeault *et al*, 2005). Only few pancreatic carcinomas are detected early enough to allow curative treatment. Current chemotherapeutic regimens are ineffective and do not prolong patient survival significantly (Haller, 2003). This explains the urgent necessity to identify the molecular alterations in pancreatic carcinomas in order to develop new treatment strategies for this neoplasm.

The transcription factor NF-*κ*B is involved in inflammatory and innate immune responses (Li and Verma, 2002). It is assembled as a hetero- or homodimer from the structurally closely related subunits RelA/p65, c-Rel, RelB, p50 and p52 (Karin *et al*, 2002). Nuclear factor-*κ*B is sequestered and inactivated in the cytoplasm by binding to one of the specific inhibitors, I*κ*B*α*, *β* or *ε*. Upon phosphorylation of the inhibitor by I*κ*B kinase (IKK), NF-*κ*B is released, translocates into the nucleus and binds to *κ*B sites in the promoters of target genes to exert its transcriptional activity (Karin *et al*, 2004). Deregulated IKK and NF-*κ*B activity contribute to tumorigenesis by triggering antiapoptotic and proproliferative responses (Rayet and Gelinas, 1999; Baldwin, 2001). Furthermore, activation of IKK and NF-*κ*B may play an important role in cancer progression elicited by inflammatory cytokines (Karin and Greten, 2005).

Increasing evidence for an involvement of NF-*κ*B in pancreatic cancer has accumulated in the last several years (Algül *et al*, 2002; Sclabas *et al*, 2003), which is not surprising given the fact that inflammatory mechanisms contribute to the genesis of this disease (Farrow and Evers, 2002). Using electrophoretic mobility shift assays comparing extracts from tumours and adjacent normal tissue, Wang *et al* (1999) demonstrated an activation of NF-*κ*B in pancreatic cancers. Furthermore, they profiled a small set of pancreatic tumours immunohistochemically and detected nuclear RelA. In another study, NF-*κ*B was found to be activated in a number of pancreatic cancer cell lines, which was later linked to chemotherapy resistance (Arlt *et al*, 2001, 2003). Consequently, inhibition of NF-*κ*B activation in these cell lines using sulfasalazine or the proteasome inhibitor MG-132 resulted in their sensitisation to etoposide, doxorubicin (Arlt *et al*, 2001) and gemcitabine (Arlt *et al*, 2003). More recently, overexpression of several NF-*κ*B subunits was described in the cytoplasm as well as in the nucleus of the pancreatic adenocarcinoma cell lines Panc-1 and BxPC-3 (Chandler *et al*, 2004). Nevertheless, a unanimous link between the overexpression of NF-*κ*B subunits and NF-*κ*B activity in these cell lines could not be established so far.

We determined expression of the NF-*κ*B subunit RelA/p65 in normal pancreas parenchyma, in chronic pancreatitis, in pancreatic intraepithelial neoplasia (PanIN) and in invasive pancreatic carcinoma and correlated the expression patterns to clinicopathological tumour characteristics. Additionally, we clarified if altered RelA expression was associated with changes in NF-*κ*B activity *in vivo* by measuring expression of NF-*κ*B-regulated genes in a subset of pancreatic carcinomas.

## MATERIALS AND METHODS

### Patient characteristics

Tissue samples from 87 patients who underwent partial pancreaticoduodenectomy for either primary pancreatic adenocarcinoma (82 cases) or chronic pancreatitis (5 cases) at the Charité University Hospital between 1991 and 2000 were used in this study (Table 1). Median age of patients with pancreatic cancer was 65 years (range 39–80 years). Follow-up data were available for all patients. Within the follow-up time, 65 patients (79.3%) died after a median follow-up time of 11.5 months. Median follow-up time of patients still alive at the end point of analysis was 44.0 months.

### Immunohistochemistry

A well-characterised monoclonal RelA-antibody (sc-8008, Santa Cruz Biotechnology, Santa Cruz, CA, USA) was used on 5 *μ*m paraffin sections (Higashitsuji *et al*, 2002; Ross *et al*, 2004). After heat-induced antigen retrieval, slides were incubated with primary antibody (1 : 250) at 4°C overnight. Bound antibody was detected by a streptavidin–biotin system (BioGenex, San Ramon, CA, USA). For colour development, a Fast Red system (Sigma, Deisenhofen, Germany) was used. The slides were cover slipped after counterstaining.

### Evaluation of staining of tissue slides

Cytoplasmic as well as nuclear RelA staining was scored by applying a semiquantitative immunoreactivity scoring (IRS) system, which ranges from 0 to 12, as described (Weichert *et al*, 2004). For statistical analysis, cases exhibiting an IRS from 0 to 6 were considered as RelA ‘negative’, cases with an IRS of 8 or higher were considered as RelA ‘positive’. Cases were grouped as nuclear-positive if distinct expression of RelA in the nuclei of tumour cells (IRS>0) could be discerned.

### RNA extraction from paraffin-embedded tumour tissue

From the whole study cohort of 82 carcinomas those cases were selected for which tumour tissue blocks without normal pancreatic parenchyma, lymph node parenchyma and duodenal wall were available. In addition, the tumour tissue blocks selected must contain at least 70% of pure carcinoma tissue, the remaining up to 30% consisting only of fat and/or connective tissue. To rule out a significant bias by variations in the density of inflammatory infiltrates, the amount of granulocytes, lymphocytes and desmoplastic stroma reaction were scored on H&E slides. None of these parameters correlated with expression of any of the genes investigated (data not shown).

The strict selection criterions were fulfilled by only 11 out of our 82 cases. From these cases, 10 *μ*m-thick paraffin sections were cut from the tissue blocks and RNA was isolated using a High Pure RNA Paraffin Kit (Roche, Mannheim, Germany). Briefly, after deparaffinisation using xylene, the tissue was homogenised by an overnight incubation with proteinase K and the nucleic acids were purified via spin-columns. Subsequently, residual DNA was digested by DNAse treatment.

### Quantitative PCR

One microgram of total RNA was reverse transcribed using AMV reverse transcriptase from Roche. Quantitative real-time PCR (TaqMan™) using Assay on Demand (number given in parentheses) from Applied Biosystems (Darmstadt, Germany) was employed to quantify the expression of RELA (Hs00153294_m1), NFKBIA (Hs00153283_m1), CCND2 (Hs00153380_m1), GAPDH (Hs99999905_m1), GUSB (Hs99999908_m1), B2M (Hs99999907_m1) and RPLPO (Hs99999902_m1) from cDNA samples. BCL2L1 and CCND1 expression was evaluated using the primers TGGAACTCTATGGGAACAATGCA (forward)/TCAGGAACCAGCGGTTGAAG (reverse) and TCGTGGCCTCTAAGATGAAGGA (forward)/GATGGAGCCGTCGGTGTAGAT (reverse), respectively. All PCRs were performed in triplicates in a 25-*μ*l reaction volume containing 2.5 *μ*l of total cDNA on an ABI Prism 7900 HT Sequence Detection System and using the following PCR conditions: 2 min at 50°C, 10 min at 95°C, followed by 40 cycles of 95°C for 10 s and 60°C for 1 min. *C*_t_ values of RELA and NF-*κ*B target genes were standardised to the average of the *C*_t_ values of the four reference genes GAPDH, GUSB, B2M and RPLPO to obtain Δ*C*_t_ values for their relative expression.

### Statistical analysis

Statistical analyses were performed using SPSS 12.0 and GraphPad Prism 4.0. Fisher's exact test and *χ*^2^ test for trends, Kaplan–Meier curves (log-rank test) and Cox regression were applied. Two-year survival rates were extracted from Kaplan–Meier survival tables. Differences in mRNA gene expression in dependence of nuclear RelA protein expression were checked with a *t*-test. Correlation of qPCR data for gene expression was performed by Pearson correlation and linear regression analyses. For correlation analyses involving RELA expression, two outlier samples were omitted, thus reducing the total sample size to nine.

## RESULTS

### Expression patterns of RelA in non-neoplastic pancreatic tissue

**Figure 1:**
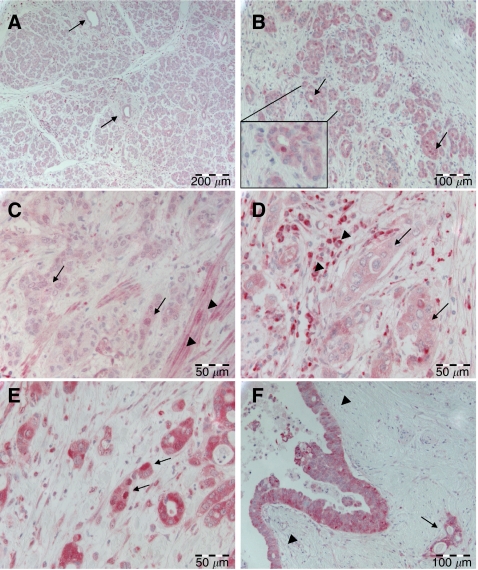
RelA/p65 expression in pancreatic tissue. (**A**) Weak cytoplasmic RelA expression in pancreatic acinar parenchyma and in pancreatic ducts (arrows). (**B**) Ductular proliferates in chronic pancreatitis with weak cytoplasmic but focal nuclear (arrows) RelA expression. Inset: Higher magnification of one duct with nuclear RelA positivity. (**C**) Ductal adenocarcinoma (arrows) with weak cytoplasmic RelA expression. Note smooth muscle cells in the tumour vicinity exhibiting nuclear RelA positivity (arrowheads). (**D**) Ductal adenocarcinoma (arrows) with moderate cytoplasmic but no nuclear RelA expression. Note strong cytoplasmic and nuclear RelA positivity in adjacent inflammatory cells (arrowheads). (**E**) Ductal adenocarcinoma with strong cytoplasmic and nuclear (arrows) expression of RelA. (**F**) PanIN III with strong cytoplasmic positivity for RelA (arrowheads). Note an invasive gland in the vicinity (arrow).

Normal acinar pancreas parenchyma as well as non-transformed ductal epithelium showed weak cytoplasmic positivity but no nuclear expression of RelA (Figure 1A). Smooth muscle cells of vessel walls and duodenal walls were weakly positive for RelA in the cytoplasm and nuclei (Figure 1C). Occasionally, RelA expression could be observed in the nuclei of stromal cells. Inflammatory infiltrate in both non-neoplastic and tumour tissue partially exhibited both strong cytoplasmic and nuclear staining (Figure 1D).

Ductular proliferates in chronic pancreatitis showed an elevated cytoplasmic expression of RelA when compared to unaffected parenchyma, with four out of six cases scoring as cytoplasmic positive for RelA. In addition, in three of the five cases investigated, occasional nuclear RelA staining was observed (Figure 1B).

### Expression patterns of RelA in ductal pancreatic adenocarcinoma

Cytoplasmic and nuclear overexpression of RelA was evident in a subset of pancreatic adenocarcinomas. However, the extent of overexpression in the majority of cases was not homogenous throughout the tissue, with some tumours exhibiting strong expression in certain clusters of tumour cells while other tumour areas displayed only weak positivity. Forty-two out of 82 adenocarcinomas (51.2%) showed cytoplasmic overexpression in neoplastic ducts (Figure 1D and E), the remaining cases were RelA-negative (Figure 1C). Additionally, 37 (45.1%) pancreatic carcinomas revealed a distinct nuclear positivity (Figure 1E). Thirty out of the 42 (81.1%) cases with cytoplasmic overexpression revealed nuclear overexpression as well (*P*<0.001) (Table 1). Cytoplasmic RelA overexpression was observed in high-grade pancreatic intraepithelial neoplasia (PanIN III), in the vicinity of invasive tumour (Figure 1F). Mesenchymal cells of desmoplastic tumour stroma exhibited nuclear RelA expression in a minority of cells as well.

### Correlation of cytoplasmic and nuclear RelA overexpression with clinicopathological factors and survival

Neither cytoplasmic nor nuclear expression patterns of RelA showed a statistically significant correlation with age and grade of differentiation (Table 1). Concerning tumour stage and nodal status, a strong trend towards higher cytoplasmic and nuclear RelA expression levels was observed for locally more advanced and nodal-positive tumours (Table 1).

**Figure 2:**
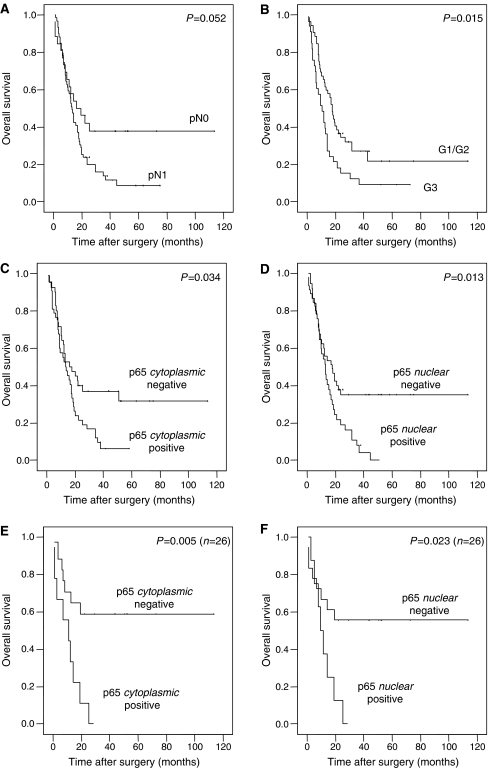
Kaplan–Meier survival curves in dependence of clinicopathological factors and RelA/p65 expression patterns. Overall survival dependent on nodal status (**A**) and grade (**B**). Overall survival dependent on cytoplasmic (**C** and **E**) and nuclear (**D** and **F**) RelA overexpression for the whole study cohort (**C** and **D**) as well as for the subgroup of node-negative patients (**E** and **F**).

Regarding patient survival, high cytoplasmic as well as high nuclear expression of RelA was significantly associated with decreased survival time (Table 2 and Figure 2). Patients with cytoplasmic negativity for RelA in the tumour survived for a median time of 16.3 month as opposed to a median survival time of 13.3 month in the group of patients whose tumours showed high RelA expression in the cytoplasm (*P*=0.034; Table 2 and Figure 2). In the group of patients with nuclear RelA positivity, the median survival time was lowered to 13.1 months compared to 17.7 months median survival for those patients without nuclear RelA expression (*P*=0.013; Table 2 and Figure 2). The estimated 2-year survival rates for patients without cytoplasmic or nuclear RelA positivity were 41 and 40%, while 2-year survival rates for patients with strong cytoplasmic or nuclear RelA expression were 22 and 20%, respectively. These survival differences were even more pronounced in the subgroup of node-negative patients showing a survival advantage for patients without cytoplasmic RelA expression (*n*=17, median survival time not reached) compared to patients with high cytoplasmic RelA expression (*n*=9, median survival time: 12.0 months) (*P*=0.005; Table 2 and Figure 2). For nuclear RelA, in this subgroup, median survival of patients with positivity in the tumour was reduced to 10.1 months while the median survival time was not reached in the remaining patients without nuclear RelA overexpression (*P*=0.023, Table 2). In multivariate survival analyses of the whole study cohort with inclusion of nodal status, grade and alternatively cytoplasmic or nuclear RelA expression, no independent prognostic impact of RelA positivity was observed (cytoplasmic: *P*=0.235, nuclear: *P*=0.120). However, in the subgroup of nodal-negative cases (*n*=26) an exploratory multivariate survival analysis with inclusion of grade and cytoplasmic RelA positivity showed RelA overexpression as a significant prognosticator (relative risk=3.49, *P*=0.020). Likewise, a similar trend was observed for nuclear positivity (*P*=0.079).

### Correlation of RelA expression with NF-*κ*B target gene mRNA expression

In the subset of tumours suited for quantitative PCR analyses, we profiled the mRNA expression of RELA (encoding for RelA/p65) and a number of NF-*κ*B target genes, such as BCL2L1 (Bcl-x_L_), CCND1 (Cyclin D1), CCND2 (Cyclin D2) and NFKBIA (I*κ*B*α*). Since NFKBIA transcription is assumed to be almost exclusively regulated by NF-*κ*B and its mRNA is also very rapidly degraded, NFKBIA has been proposed as a good surrogate marker for NF-*κ*B transcriptional activity (Emmerich *et al*, 1999).

Since intensity of cytoplasmic immunohistochemical staining was high in all cases but one, valid statistical correlations with target gene expression were not feasible. Nuclear immunohistochemical staining was scored as ‘positive’ in seven and ‘negative’ in four cases. Nuclear positivity was associated with slightly higher mean mRNA expression levels of BCL2L1 (Δ*C*_t_ 1.55 *vs* 1.75) and CCND1 (Δ*C*_t_ 2.75 *vs* 2.97), but not of CCND2 (Δ*C*_t_ 1.64 *vs* 1.06) and NFKBIA (Δ*C*_t_ −1.91 *vs* −1.56). None of these differences reached statistical significance. Since evaluation of nuclear positivity can be difficult to determine and statistics were compromised due to the small sample size and the fact that one variable was nominally scaled, we hitherto decided to correlate RELA expression levels with NF-*κ*B target gene expression.

**Figure 3:**
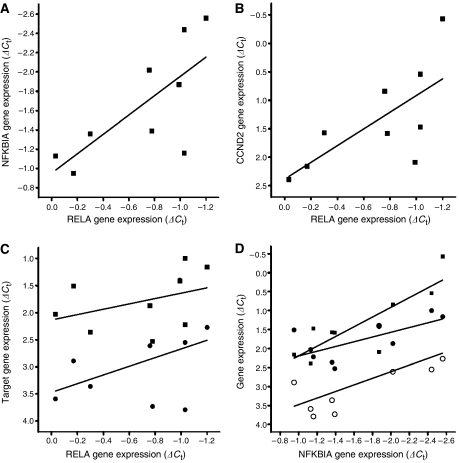
Correlation of RELA expression with NF-*κ*B target gene expression. Higher levels of RELA expression, as indicated by decreasing Δ*C*_t_ values, were significantly associated with higher expression of (**A**) NFKBIA (*r*^2^=0.4816, *P*<0.05) and (**B**) CCND2 (*r*^2^=0.5225, *P*<0.05). (**C**) A trend that higher expression levels of CCND1 (circles) and BCL2L1 (squares) correlated with higher RELA expression levels was observed as well. (**D**) NFKBIA expression correlated positively with CCND2 (squares, *r*^2^=0.7096, *P*<0.01), BCL2L1 (closed circles, *r*^2^=0.4635, *P*<0.05) and CCND1 (open circles, *r*^2^=0.4196, *P*=0.059) expression (see text).

Higher RELA gene expression was statistically significantly associated with higher NFKBIA (*r*^2^=0.4816, *P*<0.05, Figure 3A) and CCND2 expression levels (*r*^2^=0.5225, *P*<0.05, Figure 3B). Moreover, trends for a correlated expression of RELA and BCL2L1 as well as CCND1 could be observed, but failed to reach statistical significance (*r*^2^=0.1504, *P*>0.05 and *r*^2^=0.2468, *P*>0.05, respectively, Figure 3C). Considering that the expression of NFKBIA was linked with RELA expression, we sought to countercheck the observed relationships by correlating the expression of NFKBIA with other NF-*κ*B target genes. Interestingly, the expression of NFKBIA and the expression of CCND2 and BCL2L1 were associated in a statistically significant manner (*r*^2^=0.7096, *P*<0.01 and *r*^2^=0.4635, *P*<0.05, respectively, Figure 3D). CCND1 expression was correlated as well, but failed to reach statistical significance (*r*^2^=0.4196, *P*=0.059, Figure 3D). Thus, high RELA expression was linked to elevated expression of a number of NF-*κ*B target genes, first and foremost NFKBIA, arguing for simultaneous overexpression of RELA and an activation of the NF-*κ*B pathway. However, the small sample size did not permit to obtain statistically significant correlations for all genes investigated.

## DISCUSSION

Nuclear factor-*κ*B is centrally involved in tumorigenesis and tumour progression in various types of cancer. Here, we found the NF-*κ*B subunit RelA to be overexpressed in roughly half of a large set of pancreatic adenocarcinomas analysed. Overexpression of cytoplasmic and/or nuclear RelA in tumour tissue has been previously observed in larger study cohorts of gastric (Sasaki *et al*, 2001; Yamanaka *et al*, 2004; Cao *et al*, 2005; Lee *et al*, 2005), prostate (Fradet *et al*, 2004; Ismail *et al*, 2004; Ross *et al*, 2004; Shukla *et al*, 2004; Sweeney *et al*, 2004), endometrial (Pallares *et al*, 2004), hepatocellular (Tai *et al*, 2000) and oral (Nakayama *et al*, 2001) as well as cervical (Nair *et al*, 2003) carcinoma.

Reports on RelA expression in pancreatic cancer are sparse. In one study, Vimalachandran *et al* (2005) reported nuclear positivity for RelA in 23 out of 40 (57%) pancreatic cancers as detected by immunohistochemistry on tissue microarrays with an antibody directed against activated RelA. This percentage matches our results on nuclear protein expression in carcinomas. However, the authors also observed relevant cytoplasmic expression of ‘activated’ RelA in benign ducts, a finding we could not confirm.

We observed high cytoplasmic RelA expression in 42 out of 82 cases, and high nuclear RelA expression in 37 out of 82 cases, with 30 cases staining positive in both subcellular localisations and 33 cases being completely negative. Thus, cytoplasmic RelA overexpression in 81.1% of the cases was associated with nuclear positivity. One may speculate that cytoplasmic RelA overexpression is frequently and regularly accompanied by enhanced nuclear translocation and that only due to limitations of the immunohistochemical method nuclear translocation could not be seen in all cases. We assume that the very good correlations seen for cytoplasmic RelA expression indicate that it might be an even better ‘indirect’ marker for nuclear RelA amount than nuclear positivity, which is sometimes difficult to determine, itself. However, as translocation of RelA into the nucleus is a required step in the NF-*κ*B activation cascade, the nuclear localisation that was evident in 45.1% of cases can be seen as a first sign of NF-*κ*B activation. Such NF-*κ*B activation in tissue of pancreatic adenocarcinomas was previously noted by Wang *et al* (1999) and Liptay *et al* (2003). Wang *et al* (1999) observed constitutive activation of NF-*κ*B signaling in 14 out of 20 pancreatic adenocarcinomas and in 9 of 11 human pancreatic tumour cell lines. In contrast, Chandler *et al* (2004) could not unambiguously correlate overexpression of RelA with activation of NF-*κ*B signaling in two pancreatic adenocarcinoma cell lines. We show here that increased expression of RELA was correlated with higher levels of NF-*κ*B target gene expression, indicative of an activation of the NF-*κ*B pathway *in vivo*. Also, this report is the first to describe a method to reliably measure NF-*κ*B activation in archival tumour tissue.

Important NF-*κ*B target genes in cancer comprise antiapoptotic genes (Barkett and Gilmore, 1999), genes involved in angiogenesis (Aggarwal, 2004), and genes involved in the determination of invasiveness and the potential to metastasise (Fujioka *et al*, 2003a, 2003b). Consequently, NF-*κ*B inhibition by transfection with a mutated I*κ*B*α* leads to a repression of tumorigenic potential (Fujioka *et al*, 2003b), angiogenesis (Xiong *et al*, 2004) and metastatic potential (Fujioka *et al*, 2003a) of pancreatic adenocarcinoma cell lines in xenograft mouse models.

The cause of NF-*κ*B activation in pancreatic adenocarcinomas is the focus of intense investigative efforts (Sclabas *et al*, 2003). It is conceivable that K-Ras mutations, which are frequently present in pancreatic adenocarcinomas (Hruban *et al*, 1993) lead to an activation of NF-*κ*B transcriptional activity (Finco *et al*, 1997). Alternatively, NF-*κ*B might be induced via EGFR-mediated pathways, since EGFR was found to be overexpressed in 30–50% of pancreatic adenocarcinomas (Korc *et al*, 1986), and EGFR activation was proposed to lead to NF-*κ*B activation in cell lines (Habib *et al*, 2001).

The actual reasons for the overexpression of RelA in pancreatic cancer are not clear. Our data hint at enhanced transcriptional activity, as RelA protein expression is paralleled by enhanced mRNA expression. The deregulation of RelA might be caused by altered intracellular signal transduction or chromosomal overrepresentation of the respective gene locus. And in fact RELA amplification has been described in various solid tumours (Rayet and Gelinas, 1999), but not in ductal pancreatic adenocarcinomas (Schleger *et al*, 2000).

Patients with RelA-positive tumours had a significantly shortened overall survival. This observation was even more pronounced in the clinically important subgroup of nodal-negative patients, potentially amenable for curative surgical treatment. However, owing to the small case numbers in this subgroup analysis, these results need to be confirmed in a larger study cohort before draw any final conclusions. An unavoidable limitation of our study is that only patients with resectable tumours were included. Thus, it is not finally clear whether our results apply to patients with advanced non-resectable pancreatic carcinomas, as well.

Still, these findings may help to individualise treatment and to identify patients, which may profit exceptionally from NF-*κ*B-blocking agents.

The prognostic value of RelA is in concordance with studies on prostate cancer, in which both nuclear and cytoplasmic RelA overexpression were linked to disease progression (Fradet *et al*, 2004; Ross *et al*, 2004). In gastric adenocarcinoma, the prognostic impact of RelA expression appears to be somewhat conflictive, as overexpression was reported both to be an indicator of adverse (Sasaki *et al*, 2001; Yamanaka *et al*, 2004) and favourable (Lee *et al*, 2005) patient prognosis.

This study provides the missing translational link for the accumulating evidence obtained from small sets of tumour samples and from functional studies in tumour cell lines that NF-*κ*B activation plays a prominent role in pancreatic cancer development and progression. This is further emphasised by the suggestion of a hitherto unreported link between overexpression of an NF-*κ*B subunit and NF-*κ*B activation, and should lead to increased efforts for the development and use of NF-*κ*B inhibitory drugs to treat pancreatic cancer.

Importantly, the description of a significant prognostic marker is exceedingly rare in pancreatic cancer. The observation that patients overexpressing RelA show a dramatically decreased survival time may allow stratifying patients into two subgroups: one subgroup is likely to benefit from NF-*κ*B-inhibiting agents and another subgroup which has a relatively good survival perspective irrespective of such a treatment, potentially because resistance to conventional chemotherapeutics due to NF-*κ*B activation has not occurred yet (Arlt *et al*, 2001, 2003). This possibility should be considered when clinical trials with NF-*κ*B-inhibiting agents are being planned.

## Figures and Tables

**Table 1:** Distribution of clinicopathological characteristics in the study cohort and correlation with cytoplasmic and nuclear RelA expression

	**All cases**	**RelA cytoplasmic negative**	**RelA cytoplasmic positive**	***P*-values**	**RelA nuclear negative**	**RelA nuclear positive**	***P*-values**
*All cases*	82	40 (48.8%)	42 (51.2%)		45 (54.9%)	37 (45.1%)	
							
*Age (years)*							
⩽65	42 (51.2%)	20 (47.6%)	22 (52.4%)	1.000^a^	23 (54.8%)	19 (45.2%)	1.000^a^
>65	40 (48.8%)	20 (50%)	20 (50%)		22 (55%)	18 (45%)	
							
*Tumour stage*							
T1	1 (1.2%)	1 (100%)	0 (0%)	0.062^b^	1 (100%)	0 (0%)	0.050^b^
T2	30 (36.6%)	17 (56.7%)	13 (43.3%)		19 (63.3%)	11 (36.7%)	
T3	48 (58.5%)	22 (45.8%)	26 (54.2%)		25 (52.1%)	23 (47.9%)	
T4	3 (3.7%)	0 (0%)	3 (100%)		0 (0%)	3 (100%)	
							
*Nodal status*							
N0	26 (31.7%)	17 (65.4%)	9 (34.6%)	0.057^a^	18 (69.2%)	8 (30.8%)	0.097^a^
N1	56 (68.3%)	23 (41.1%)	33 (58.9%)		27 (48.2%)	29 (51.8%)	
							
*Lymph vessel invasion*							
L0	54 (65.9%)	22 (40.7%)	32 (59.3%)	0.062^a^	28 (51.9%)	26 (48.1%)	0.490^a^
L1	28 (34.1%)	18 (64.3%)	10 (35.7%)		17 (60.7%)	11 (39.3%)	
							
*Blood vessel invasion*							
V0	71 (86.6%)	34 (47.9%)	37 (52.1%)	0.753^a^	39 (54.9%)	32 (45.1%)	1.000^a^
V1	11 (13.4%)	6 (54.5%)	5 (45.5%)		6 (54.5%)	5 (45.5%)	
							
*Grade*							
G1	8 (9.8%)	2 (25%)	6 (75%)	0.939^b^	4 (50%)	4 (50%)	0.205^b^
G2	43 (52.4%)	25 (58.1%)	18 (41.9%)		28 (65.1%)	15 (34.9%)	
G3	31 (37.8%)	13 (41.9%)	18 (58.1%)		13 (41.9%)	18 (58.1%)	
							
*RelA nuclear*							
Negative	45 (54.9%)	33 (73.3%)	12 (26.7%)	<0.001^a^	—	—	
Positive	37 (45.1%)	7 (18.9%)	30 (81.1%)		—	—	

a Fisher's exact test.

b *χ*^2^ test for trends.

**Table 2:** Patient survival in dependence of several clinicopathological factors and cytoplasmic/nuclear RelA expression

	**Cases**	**Events**	**Median survival (months)**	**Standard error**	**Log-rank test (*P*-values)**
*Age at diagnosis (years)*					
⩽65	42	34	12.0	1.9	0.229
>65	40	31	17.7	2.0	
					
*Tumour stage*					
T1/T2	31	26	16.3	2.9	0.895
T3/T4	51	39	13.3	3.1	
					
*Nodal status*					
N0	26	16	16.3	5.9	0.052
N1	56	49	13.1	1.6	
					
*Grade*					
G1/G2	51	37	18.0	1.5	0.015
G3	31	28	10.1	2.6	
					
*RelA cytoplasmic*					
Negative	40	26	16.3	6.9	0.034
Positive	42	39	13.3	1.9	
					
*RelA cytoplasmic (node negative)*					
Negative	17	7	Not reached	—	0.005
Positive	9	9	12.0	2.7	
					
*RelA nuclear*					
Negative	45	29	17.7	4.4	0.013
Positive	37	36	13.1	1.8	
					
*RelA nuclear (node negative)*					
Negative	18	8	Not reached	—	0.023
Positive	8	8	10.1	2.8	
